# Supplementary material for: A genus in the bacterial phylum Aquificota appears to be endemic to Aotearoa-New Zealand
Source: Nat Commun. 2024 Jan 2;15:179. doi: 10.1038/s41467-023-43960-2 (PMC10762115; doi:10.1038/s41467-023-43960-2)
Supplement: Supplementary file 1 — Supplementary Information [file 41467_2023_43960_MOESM1_ESM.pdf]

SUPPLEMENTARY INFORMATION

A genus in the bacterial phylum Aquificota appears to be endemic to Aotearoa-New Zealand.

Jean F. Power[1], Carlo R. Carere[2], Holly E. Welford[3], Daniel T. Hudson[4], Kevin C. Lee[5], John W. Moreau[6], Thijs J. G. Ettema[7], Anna-Louise Reysenbach[8], Charles K. Lee[1], Daniel R. Colman[9], Eric S. Boyd[9], Xochitl C. Morgan[4,10], Ian R. McDonald[1], S. Craig Cary[1*], & Matthew B. Stott[3*]

[1]Thermophile Research Unit, Te Aka Mātuatua | School of Science, Te Whare Wānanga o Waikato | University of Waikato, Hamilton 3240, Aotearoa-New Zealand
[2]Te Tari Pūhanga Tukanga Matū | Department of Chemical and Process Engineering, Te Whare Wānanga o Waitaha | University of Canterbury, Christchurch 8140, Aotearoa-New Zealand
[3]Te Kura Pūtaiao Koiora | School of Biological Sciences, Te Whare Wānanga o Waitaha | University of Canterbury, Christchurch 8140, Aotearoa-New Zealand
[4]Te Tari Moromoroiti me te Ārai Mate | Department of Microbiology and Immunology, Te Whare Wānanga o Ōtākou | University of Otago, Dunedin 9054, Aotearoa-New Zealand
[5]Te Kura Pūtaiao | School of Science, Te Wānanga Aronui o Tāmaki Makau Rau | Auckland University of Technology, Auckland 1010, Aotearoa-New Zealand
[6]School of Geographical & Earth Sciences, University of Glasgow, Glasgow, G12 8QQ, UK
[7]Laboratory of Microbiology, Wageningen University & Research, 6708 WE Wageningen, the Netherlands
[8]Department of Biology, Portland State University, Portland, Oregon 97207, USA
[9]Department of Microbiology and Cell Biology, Montana State University, Bozeman, MT 59717, USA
[10]Department of Biostatistics, Harvard T. H. Chan School of Public Health, Boston, MA 02115, USA

***Correspondence:**
Matthew Stott - matthew.stott@canterbury.ac.nz
Craig Cary - caryc@waikato.ac.nz

The following Supplementary Information document contains:

1. Supplementary Notes 1-4
2. Supplementary Figures S1-S7
3. Supplementary References

SUPPLEMENTARY NOTES

Supplementary Note 1:
Genome annotation of *V. stagnispumantis* CP.B2^T

Forty-seven scaffolds from the draft genome sequence of CP.B2^T were analysed through the Integrated Microbial Genomes annotation pipeline v4.16.4 (IMG Taxon ID 2799112217, GOLD Analysis ID Ga0311387)[1]. The CP.B2^T genome is also accessible in GenBank (GCA_026108055.1). Estimated total size was 1.6 Mbp, with 29.6 % mol G+C. The number of protein coding genes was 1,707, with 1,409 of these having predicted function. Two 16S rRNA genes were detected. A second copy of the CP.B2^T genome exists in GOLD (Analysis ID Ga0170441) and IMG (Taxon ID 2724679818), sequenced directly from the culture collection DSMZ (DSM 18763) which was used to corroborate annotation. Detailed annotation on carbon assimilation, electron transport, sulfur, nitrogen and arsenic metabolisms, transmembrane transportation, cytosolic pH moderation, and comparison of Hydrogenothermaceae can be found below, with a full list of genes annotated with predicted function outlined in Supplementary Data 11.

Carbon assimilation

Presence of the Type 1 reductive TCA (rTCA) cycle was evident by the annotation of ATP-citrate lyase (ACL; gene *aclAB*), succinate dehydrogenase/fumarate reductase (*sdhABC*), and 2-oxoglutarate:ferredoxin oxidoreductase (*korAB*) genes[2]. Citryl-coA synthetase (*ccsAB*) and 2-oxoglutarate carboxylase (*cfiAB*), necessary for the alternate Type II rTCA cycle used by the Aquificaceae, were not present[2,3]. ACL is thought to have been acquired by the Hydrogenothermaceae through horizontal gene transfer[2], initially formed from a fusion of citryl-coA lyase (*ccl*) and citryl-coA synthetase (*ccsAB*), both used in the older Type II cycle. There was also no evidence of citrate synthase (*gltA*), found in many autotrophic bacteria, which can be used in both the oxidative TCA cycle for the production of citrate and the reversed oxidative cycle (roTCA) to fix carbon dioxide[4]. An entire Embden-Meyerhof-Parnas (EMP) pathway for glycolysis/gluconeogenesis was annotated[5], with no evidence found of the alternate Entner-Doudoroff (ED) pathway or the oxidative branch of the pentose phosphate pathway (oxPPP), traditional sources of NADPH for cell metabolism[6]. A non-phosphorylating glyceraldehyde-3-phosphate dehydrogenase gene (*gapN*) was annotated, which can produce

NADPH by irreversibly oxidising glyceraldehyde-3-phosphate (GAP) straight to 3-phosphoglycerate (3-PG) in the EMP pathway[6]. There was also no evidence of genes for carbon monoxide dehydrogenase, or ribulose-bisphosphate carboxylase (RuBisCo) from the Calvin-Benson-Bassham cycle.

Electron transport

The genome had 13 subunits of the proton-translocating NADH:ubiquinone oxidoreductase (*nuoA-N*) for complex I of the electron transport chain. Succinate dehydrogenase (*sdhABC* and *frdB*) was annotated for complex II. Three subunits for the cytochrome bc1 complex (cytochrome c oxidoreductase) were encoded for complex III (*petABC*), with cytochrome bd being the respiratory terminal oxidase (*cydAB*; complex IV). Research has shown that cytochrome bd increases expression in response to a range of environmental stressors, including pH and temperature extremes[7]. The genome had membrane-bound F-type ATPases (*atpA-H*) for complex V.

Sulfur, nitrogen & arsenic

The only gene found in the CP.B2^T genome from the SOX pathway (*soxD*) for sulfur/thiosulfate oxidation is a subunit in cytochrome c of the electron transport chain, and may also be involved in arsenic cycling[8]. Cytochrome c is reduced as an intermediate between complexes III and IV[9], and a proton gradient is created for ATP synthesis. There was no evidence of sulfite dehydrogenase (*sorAB* or *soeA*) in the genome, even though genes were present for the biosynthesis of the cofactor molybdopterin[10]. No further genes from other sulfur-metabolising pathways including sulfur oxygenase reductase (SOR), thiosulfate dehydrogenase (*tqoAB*), dissimilatory sulfite reductase (*dsrABC*), and heterodisulfide reductase subunits *hdrC1* and *hdrB2*[11] were detected. Nitrogen assimilation via the uptake of ammonia by glutamine synthetase (*glnA*) and glutamate synthase (*gltBD*) was noted, along with nitronate monooxygenase (NMO) which putatively generates nitrite from nitroalkane. No genes associated with nitrogen-dissimilatory pathways were identified. There was no evidence of genes encoding nitric oxide reductase (*norB*) for denitrification, which is commonly found in other Hydrogenothermaceae. Congruently, CP.B2^T was the only Hydrogenothermaceae analysed to not encode a nicotinamidase gene (*pncA*). While the arsenic resistance operon *arsRBC* was annotated in the CP.B2^T genome, there was no indication of the arsenic ABC transporter ATPase gene (*arsA*; as part of the more complex *arsRDABC* operon)[12].

Additionally, no putative genes involved with dissimilatory arsenic metabolism were found, such as arsenate reductases (*arrAB*), including those found in *Pyrobaculum arsenaticum*[13] and *Geobacter lovleyi*[14], and arsenite oxidase (*aioAB*)[15], which is prevalent in *Sulfurihydrogenibium*[16].

Transmembrane transportation

Genes associated with transport systems for the facilitated diffusion of ions across the membrane were found for sodium, iron, calcium, magnesium, iron and ammonium, with multiple transport systems found for potassium. There were also several ABC transporters for zinc, cobalt, nickel, phosphate and a range of molecules involved in cell membrane formation (e.g., phospholipids, lipopolysaccharides, and lipoproteins). While a gene for the molybdate transport system regulatory protein (*modE*) was present, the rest of the high-affinity molybdate uptake system (*modABCD*) was missing. This is contrary to the rest of the Hydrogenothermaceae. There was also one copper exporting ATPase (*copB*) encoded in the genome, whereas all other Hydrogenothermaceae analysed had a range of two to five.

Ability to moderate cytosolic pH

Two genes associated with the *aguBDAC* operon for increasing alkalinity within the cell, agmatine deiminase (*aguA*) and putrescine amidase (*aguB*), were detected in the genome. Potential for malolactic fermentation was also noted by the presence of a lactate dehydrogenase gene (*dld*). No copies of glutamate, arginine or lysine decarboxylase, urease, or tryptophanase were observed, indicating that *Venenivibrio* does not possess these well-known mechanisms to manage cell pH homeostasis[17]. However, it should be noted that all Hydrogenothermaceae family members analysed appear to have no significant differences in genomic capabilities when it comes to cytosolic pH modulation.

Hydrogenothermaceae genomes

Along with CP.B2^T, a group 2d hydrogenase was found in three *Persephonella* spp., *H. marinus* VM1^T, and *S. subterraneum* HGMK1^T. *Sulfurihydrogenibium* sp. Y03AOP1 and *S. yellowstonense* SS-5^T had no hydrogenases, and instead rely on the oxidation of reduced sulfur species[18]. An arsenite oxidase gene (*aioA*, formally *aroA* or *aoxA*)[15], which was found in four *Sulfurihydrogenibium* spp. analysed, was not annotated in the CP.B2^T genome.

Supplementary Note 2:

Growth range reassessment of *V. stagnispumantis* CP.B2^T

Moderate growth of CP.B2^T was observed from 40.9-79.7 °C (opt 70.4 °C), with limited growth noted at both 38.5 °C and 80 °C. Cells at 80 °C were not viable when subcultured to new DSMZ Medium 1149 and cultivated at 70 °C. Growth was observed between pH 4.5-8.0, with the most significant growth observed at pH 5.5-7.0 (opt pH 6.0). A reduced number of viable cells were also visualised in medium from pH 3.0-4.0 and pH 8.5. Salinity tolerance was confirmed at 0.0-8.0 % NaCl (opt 0.0-0.2 %). Cells of CP.B2^T tolerated O_2 concentrations of up to 10 % v/v and as low as 1.25 % v/v of the headspace. Growth of CP.B2^T in Champagne Pool water was determined by the presence of typical aggregates in all three preparations. Within 24 hours, growth was also observed in all hybrid Obsidian Pool medium preparations. Microscopy confirmed increased numbers of CP.B2^T cells were viable in subsequent subculturing of unaltered spring water. Both the Champagne Pool and Obsidian Pool cultures were sequentially subcultured five times to ensure growth was not a result of essential element(s) carryover. Results are summarised in Supplementary Data 12.

Supplementary Note 3:

Global search for 16S rRNA genes reported as, or closely related to, *Venenivibrio*

Nucleotide Collection (NCBI)

The Nucleotide collection (nt) of NCBI had 71,983,277 non-redundant DNA sequences at the time of analysis. Two entries were deposited for the 16S rRNA gene of *V. stagnispumantis* CP.B2^T (1506 bp in length); the first discovery of the gene in Champagne Pool, Aotearoa-New Zealand in 2007 (GenBank accession DQ989208)[19], and the characterisation of the type strain in 2008 (NR_044029)[20]. There was only one result with both ≥95 % sequence similarity and ≥95 % query coverage to the near full length 16S rRNA gene, a clone deposited in 2003 from a hot spring in Kuirau Park, Aotearoa-New Zealand (AF402979; 98.6 % sequence similarity, 1441 bp)[21]. The next most similar result across the full gene was *Sulfurihydrogenibium azorense* Az-Fu1^T (CP001229; 94.5 % sequence similarity). Six other partial entries (536-899 bp) were found with ≥98.0 % similarity, which were all clones from two separate studies of Champagne Pool; EF101539 and EF101540[19], and FN429034, FN429035, FN429036, and FN429037[22]. The next closest results to CP.B2^T were 29 clones (538-629 bp) from the same

study[23], with a similarity range of 95.2-95.7 %. These were most closely related to *S. azorense* Az-Fu1^T (98.9-99.8 %) and were from a hot spring in the Nagano Prefecture, Japan. All other results from NCBI, both full and partial length, were <95 % sequence similarity to *V. stagnispumantis* CP.B2^T.

Sequence Read Archive (NCBI)

Three samples from SRA had total k-mer counts of 1672, 3218 and 7146 that assigned to the genus *Venenivibrio* when searched using the STAT program (Supplementary Data 15), which searched 208.5 gb of metadata from a possible 12.2 petabytes (or 27.3 quadrillion bases) of open access sequence data (06/Dec/2021; https://www.ncbi.nlm.nih.gov/sra/docs/sragrowth/). One of these samples was a metagenome from a geothermal spring in Aotearoa-New Zealand that was already included in this study (P1.0019, Radiata Pool; SRR14702244). The other two (SRR15830908 and SRR15830907) were 16S rRNA gene amplicon samples from seafloor hydrothermal vents near the Baja California Peninsula, Mexico. The greatest sequence similarity to the full length 16S rRNA gene of *V. stagnispumantis* CP.B2^T found across both of these samples was 92 %, with 16 % of the gene covered by the query. The remaining samples from this SRA search (n=85) that produced k-mers assigned to *Venenivibrio* had counts that ranged between 25 and 430 (Supplementary Data 15).

SILVA database

The SILVA SSU r138.1 database contained a total of 33 entries classified to *Venenivibrio*, including the 16S rRNA gene of *V. stagnispumantis* CP.B2^T, from a total of 9,469,124 aligned rRNA sequences (Supplementary Data 13). Seven of these entries were clones originating from Aotearoa-New Zealand hot springs and all had ≥98.0 % pairwise sequence similarity to *V. stagnispumantis* CP.B2^T (GenBank accessions AF402979, EF101539, EF101540, FN429034, FN429035, FN429036, and FN429037)[19,21,22]. The remaining 25 entries composed of one isolate and 24 clones and had a similarity range of 79.3-94.7 % to *V. stagnispumantis* CP.B2^T (Supplementary Data 13). An approximate maximum-likelihood phylogenetic tree of all 33 aligned sequences clustered only the seven Aotearoa-New Zealand clones together with *V. stagnispumantis* CP.B2^T (Figure S6). SILVA identified one closest neighbour (≥95 % identity) to *V. stagnispumantis* CP.B2^T in the Ref NR database, the Aotearoa-New Zealand clone AF402979[21].

Ribosomal Database Project (RDP)

RDP (release 11_6) had a total of 3,351,829 sequences screened. Seven of these sequences matched the 16S rRNA gene sequences of *V. stagnispumantis* CP.B2^T (98.6-99.6 % sequence similarity), which were the same clones originating from Aotearoa-New Zealand that were found in the GenBank and SILVA databases (GenBank accessions AF402979, EF101539, EF101540, FN429034, FN429035, FN429036, and FN429037)[19,21,22].

Greengenes database

The latest version of the Greengenes 16S rRNA gene database v13.8 (August 2013) had 1,262,986 unique sequences. These were clustered into 203,452 and 99,321 representative OTUs at 99 % and 97 % similarity, respectively. Only one of these OTUs classified as *Venenivibrio stagnispumantis* (Greengenes OTU ID 1142935) in the 99 % representative set, with one additional sequence in the database (ID 189417) also found mapped to this cluster. Both sequences corresponded to the 16S rRNA gene sequence of the type strain, CP.B2^T (GenBank accessions DQ989208 and NR_044029). No OTU in the 97 % representative set was assigned to *Venenivibrio*, with only 25 classifying to the family Hydrogenothermaceae. From these 25 OTUs, OTU ID 32720 had 98.6 % sequence similarity to *V. stagnispumantis* CP.B2^T and the corresponding GenBank accession was AF402979, a clone found in a hot spring from Kuirau Park, Aotearoa-New Zealand[21]. The next highest similarity from the 97 % OTUs was OTU ID 242647 at 94.2 %, with all others at ≤92.7 %. OTU ID 1142935 was also mapped to OTU 32720 cluster in the 97 % reference set.

Integrated Microbial Next Generation Sequencing (IMNGS) platform

From a total of 500,048 samples (24,835,679,746 reads), 31 OTUs were found across 29 samples with ≥95 % sequence similarity to the 16S rRNA gene of *V. stagnispumantis* CP.B2^T (Supplementary Data 14). None of these OTUs had ≥99 % similarity, with only eight having ≥97 % across ≤34 % of the CP.B2^T 16S rRNA gene. One of these OTUs (with ≥97 % similarity) was sourced from a sample of sugarcane root soil in Australia (SRA run accession SRR1924223)[24], and two were from peat soil adjacent to cold temperature springs in Canada (SRR1029457 and SRR2026416)[25]. However, the abundance of these OTUs ranged from one to five reads which contributed to only ≤0.01 % of the sample communities and were present in ecosystems not conducive to supporting *Venenivibrio* populations (e.g., soils, cold temperature). The remaining five OTUs with ≥97 % similarity to CP.B2^T were sourced from

synthetic samples (Supplementary Data 14). Samples containing reads at ≥95 % sequence similarity to *Venenivibrio* which accounted for ≥0.1 % of the sample community were all from Aotearoa-New Zealand geothermal springs (*n*=10).

Integrated Microbial Genomes and Microbiomes (IMG/M) database

IMG/M had a total of 147,328 datasets at the time of analysis, including 26,097 distinct public non-redundant genomes and 83,287 metagenomic bins. Thirteen genomes were found classified to the Hydrogenothermaceae from 11 isolates and two MAGs. Two of these genomes were found assigned to the genus *Venenivibrio*: *Venenivibrio stagnispumantis* DSM 18763 (IMG Genome ID 2724679818; Gold Analysis ID Ga0170441), which was sequenced from the type strain stored in the DSMZ culture collection; and *Venenivibrio stagnispumantis* CP.B2 (IMG Genome ID 2799112217; Gold Analysis ID Ga0311387), which was sequenced by this study from the type strain stored in the original laboratory that isolated the microorganism. No other genomes in the entire collection contained genes that matched the 16S rRNA gene of *V. stagnispumantis* CP.B2^T. Likewise, no metagenome bins were found assigned to the genus. There were 20 bins classified to the Hydrogenothermaceae, using GTDB-Tk lineage, and these were either *Sulfurihydrogenibium* (*n*=11) or *Persephonella* (*n*=9).

Earth Microbiome Project (EMP) & Qiita database

No samples from the first release of the Earth Microbiome Project (EMP) had sequences that matched *Venenivibrio* in the analysis classified using SILVA taxonomy. This dataset had a total of 27,411 samples, 126,730 OTUs, and 1,754,319,647 sequence reads. The reference OTUs for this analysis were checked and two OTUs were classified to the genus *Venenivibrio* (AF402979.1.1441 and KM221400.1.1484). To confirm this result, the redbiom search function was enabled for both taxon (g__Venenivibrio) and features (OTU ID 32720 and 1142935) in the Greengenes-assigned taxonomy. Again, no hits were found. The EMP subset used to create trading cards had 155,002 unique sequences (ASVs; amplicon sequence variants) from a total of 10,000,000 reads, 2,000 samples and 95 studies. *Venenivibrio* was also not found in this subset and there were only two Hydrogenothermaceae-assigned ASVs, one found in five samples with a total of 238 reads, the second found in one sample with six reads (or observations). These six samples were from the Lost City Hydrothermal Field, Mid-Atlantic Ridge, and no significant similarity was found to the 16S rRNA gene of *V. stagnispumantis* CP.B2^T. Finally, no results were found for *Venenivibrio* within publicly available studies

(*n*=599) in the Qiita platform. A total of 276,184 samples were searched by taxon name, OTU IDs and sequence.

NCBI & Google Scholar word search

A word search for *Venenivibrio* in all NCBI databases highlighted four entries in SRA and four entries in GenBank. Two of the SRA entries belonged to whole genome sequencing of the type strain CP.B2^T from the culture collection DSMZ (DSM 18763; SRA runs SRR5889102 and SRR5889103). The other two entries were amplicon sequences added in 2020 from an unpublished study of hot spring microbial communities in SiChuan, China (SRR10580885 and SRR10580889). The highest sequence similarity found with the full length 16S rRNA gene of *V. stagnispumantis* CP.B2^T was 93.1 %, with 16 % of the gene covered. The GenBank results included three accession numbers from the type strain CP.B2^T (DQ989208, NR_044029, and EF581124)[19,20], with the fourth result from an environmental clone labelled as uncultured *Venenivibrio* sp. CCB8131 (GenBank accession KY480601). This sequence had only 78.3 % sequence similarity to *V. stagnispumantis* CP.B2^T, with 85 % of the query covered.

There were eight published manuscripts that contained the word '*Venenivibrio*' from NCBI's PubMed Central database (PMC; accessed 28/Apr/2022). These included five studies that used samples from Aotearoa-New Zealand geothermal springs[26–30], and three publications that referenced the type strain *V. stagnispumantis* CP.B2^T and/or associated characterisation[31–33]. A similar search was also conducted in Google Scholar (accessed 28/Apr/2022) which highlighted an additional nine manuscripts that referenced the type strain[34–42], plus two that reported *Venenivibrio* taxa in amplicon sequencing of Chinese hot spring and wetland microbial communities[43,44]. The first of these studies described an average of 8.7 % *Venenivibrio* in the microbial communities across 16 hot springs[43]; however, the greatest sequence similarity to CP.B2^T from all of these samples was 93.5 % over 16 % of the 16S rRNA gene. The second study reported trace amounts of *Venenivibrio* (0.03-2.15 %) in three microbial communities of a voltage-applied wetland plant[44]. The 16S rRNA gene sequences from this study were not deposited in a database for review, so this result could not be verified.

Supplementary Note 4:
Screening metagenomes for *Venenivibrio*

Similar to *V. stagnispumantis* CP.B2^T, low coverage breadth was observed from the six global metagenomes mapped to *P. hydrogeniphila* 29W^T, *S. yellowstonense* SS-5^T and *Sulfurihydrogenibium* sp. Y03AOP1 (Figure 6, Supplementary Data 18). This could be due in part to low numbers of Hydrogenothermaceae originally present in the sites analysed, enrichment strategies excluding preferred Aquificota growth conditions, or perhaps the samples contained reads from an uncharacterised close relative of the genomes tested. The three samples from Japan had the highest coverage breadth of *P. hydrogeniphila* 29W^T at 2.8-6.1 % (Supplementary Data 18).

To test the sensitivity of Kraken2 at classifying reads as *Venenivibrio*, eight mock communities (samples A-H) were created with varying concentrations of *V. stagnispumantis* CP.B2^T, *Sulfurihydrogenibium* sp. Y03AOP1, *P. hydrogeniphila* 29W^T and a selection of random genomes (Supplementary Data 20). The software correctly classified concentrations of both *V. stagnispumantis* (±0.93 %) and *Sulfurihydrogenibium* sp. Y03AOP1 (±1.39 %), but failed to classify *P. hydrogeniphila* whatsoever. Non-equivalent concentrations were reported, however, for *P. marina* EX-H1 in those mock communities with *P. hydrogeniphila*. Interestingly, the sample that consisted of *P. hydrogeniphila* reads only (sample B) returned 1.42 % of the community as *Venenivibrio*, suggesting that previous traces of *Venenivibrio* found globally in both metagenomic and amplicon samples could have been inflated by similar family members being present in these sites.

The alignment of synthetic metagenomes back onto their respective genomes did not yield any anomalies (Figure S7; Supplementary Data 21). In the three mock communities where *V. stagnispumantis* concentration was either 100, 10, or 33 %, the type strain genome was predominantly covered by each sample (98.7, 98.3, and 98.6 % respectively). Even in the samples with 1 % of the species, 83.2 % of the genome was recovered. While classification of samples with *P. hydrogeniphila* proved unreliable, this was not the case with aligning synthetic reads back onto this genome (>99.4 % coverage breadth with initial concentrations of 33, 45, or 100 %). *Sulfurihydrogenibium* sp. Y03AOP1 also had near complete coverage breadth across the genome (>99.96 %), with only <0.9 % of another species in the same genus (*S.*

yellowstonense SS-5^T) being covered. The depth of coverage from the recovered genomes varied across samples, depending on taxon concentration that comprised the initial community makeup (Supplementary Data 21). Average coverage depth of *V. stagnispumantis* was 100x in the sample with 100 % of reads from that species. This reduced to 2.3x for samples with just 1 % *V. stagnispumantis*. A similar trend was observed for coverage depth for *P. hydrogeniphila* and *Sulfurihydrogenibium* sp. Y03AOP1.

SUPPLEMENTARY FIGURES

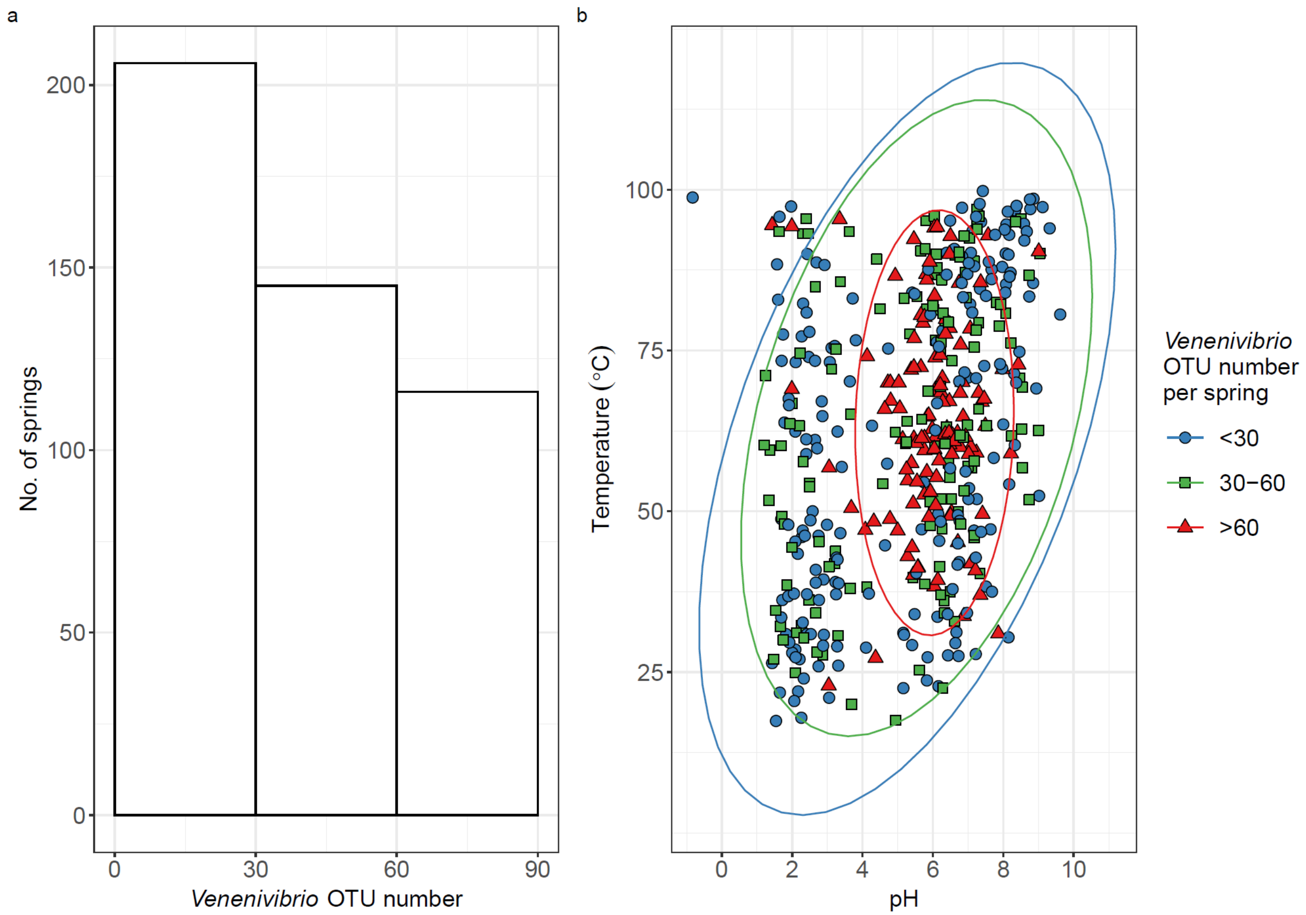

Figure S1. ***Venenivibrio*** **16S rRNA gene diversity in the Taupō Volcanic Zone (TVZ), Aotearoa-New Zealand. (a)** The number of operational taxonomic units (OTUs) found across 467 geothermal springs that assigned to the genus *Venenivibrio* are shown (post filtering; *n*=99 OTUs). **(b)** Springs (*n*=467) are plotted as a function of environmental pH and temperature conditions. The number of *Venenivibrio*-assigned OTUs in each spring is represented by blue circles (<30), green squares (30-60), or red triangles (>60), with data ellipses assuming multivariate t-distribution and a 95 % confidence interval.

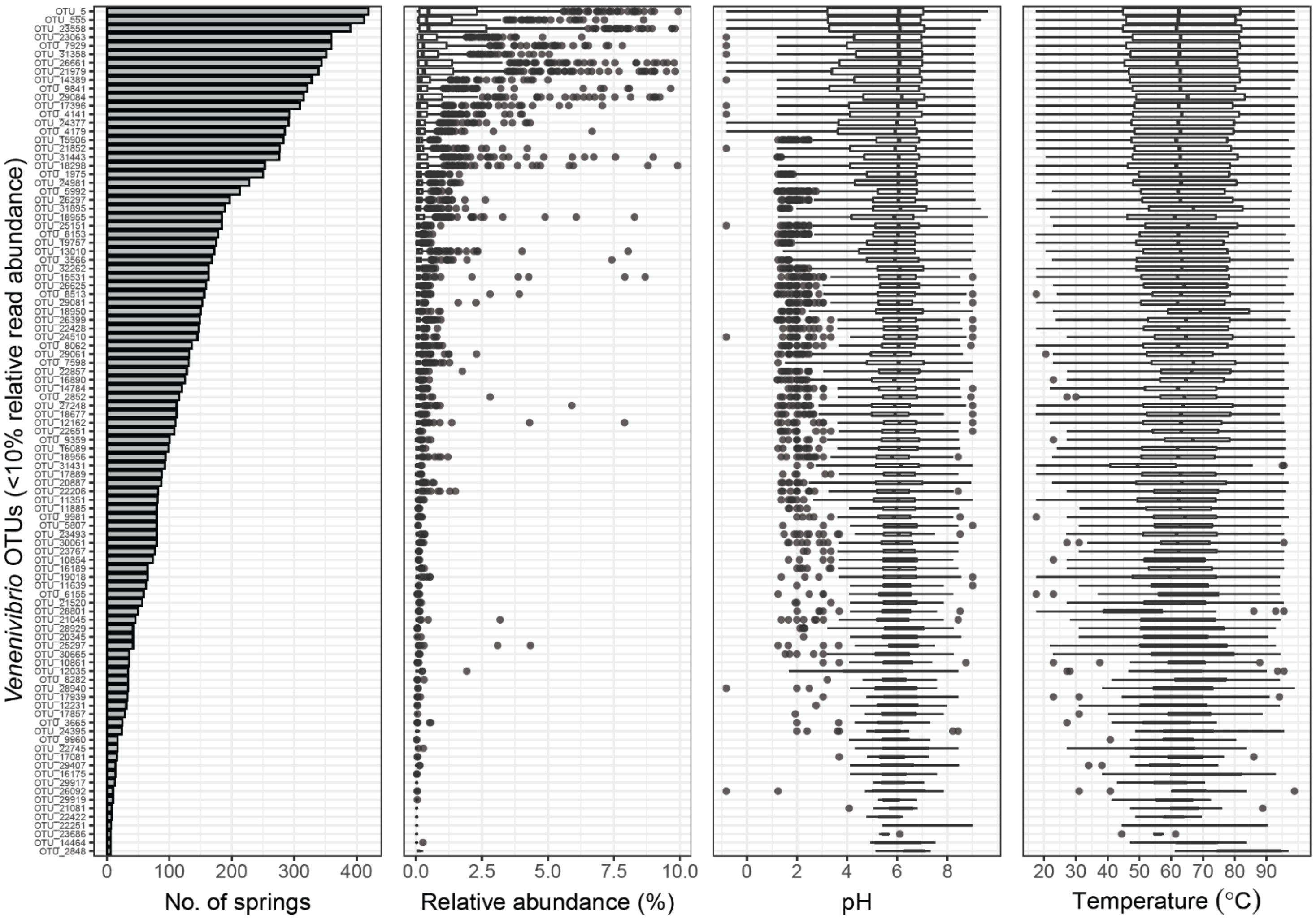

Figure S2. Prevalence, read abundance, pH, and temperature ranges of low abundance *Venenivibrio* operational taxonomic units (OTUs). Low abundance OTUs (<10 % relative abundance per spring community) that assigned to the genus *Venenivibrio* are shown (post filtering; *n*=99 OTUs). These are ordered by the number of springs where each OTU was found (i.e., prevalence). Median pH and temperature for low abundance OTUs were 6.0 (IQR 1.7) and 62.9 °C (IQR 23.4), respectively.

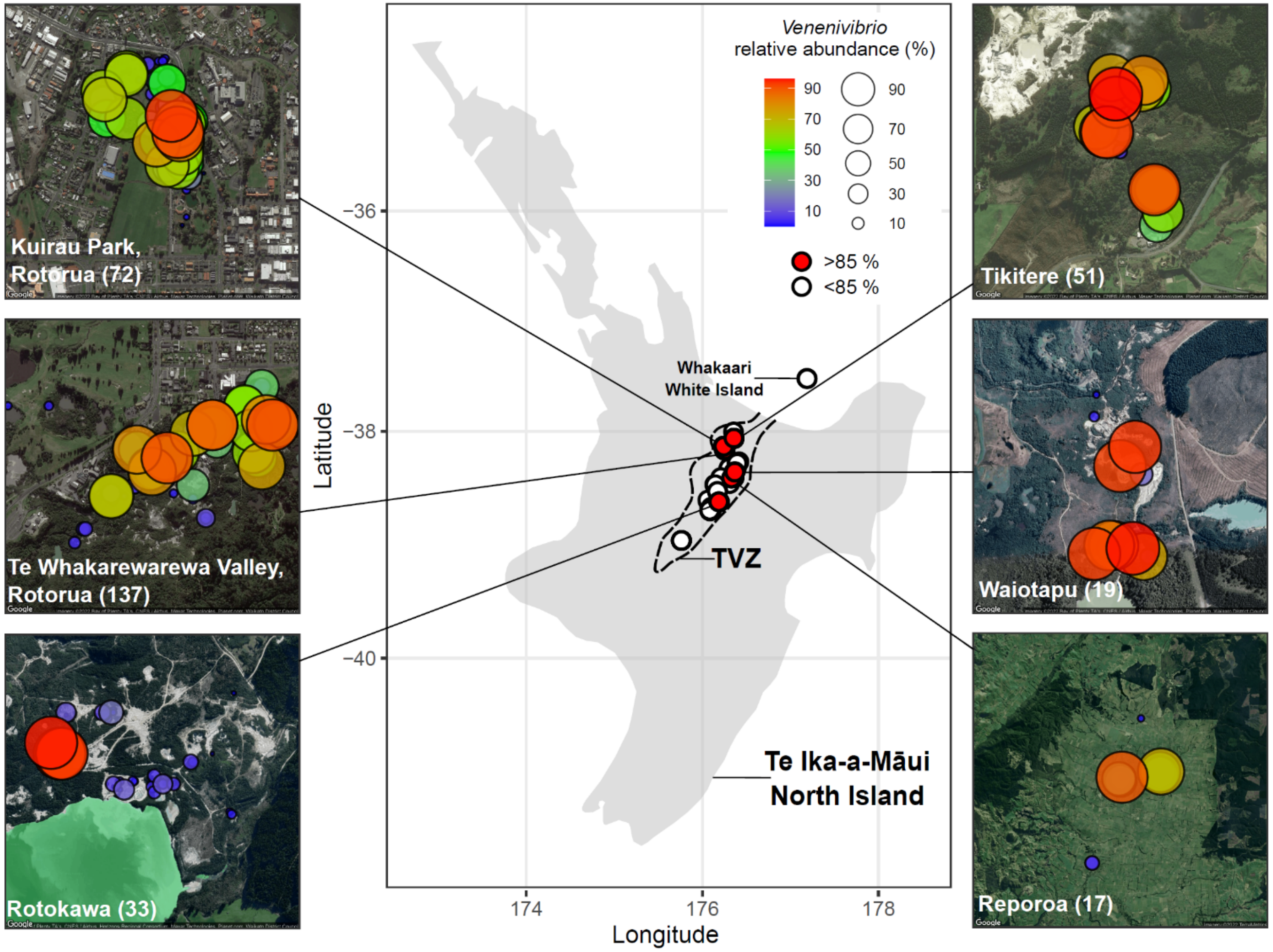

Figure S3. *Venenivibrio* hotspots in the Taupō Volcanic Zone (TVZ), Aotearoa-New Zealand. Geothermal springs containing *Venenivibrio* 16S rRNA genes (post filtering; n=467) are shown in the centre map, with springs containing ≥85 % of the microbial community (n=20) highlighted in red. These springs are also presented in their respective geothermal fields, with circles coloured and sized according to *Venenivibrio* relative read abundance per spring community. Total number of springs that contained *Venenivibrio* per geothermal field are in brackets. Median pH and temperature of these hotspots were 5.5 (IQR 0.8) and 66.0 °C (IQR 18.1), respectively. Map data ©2022 Google.

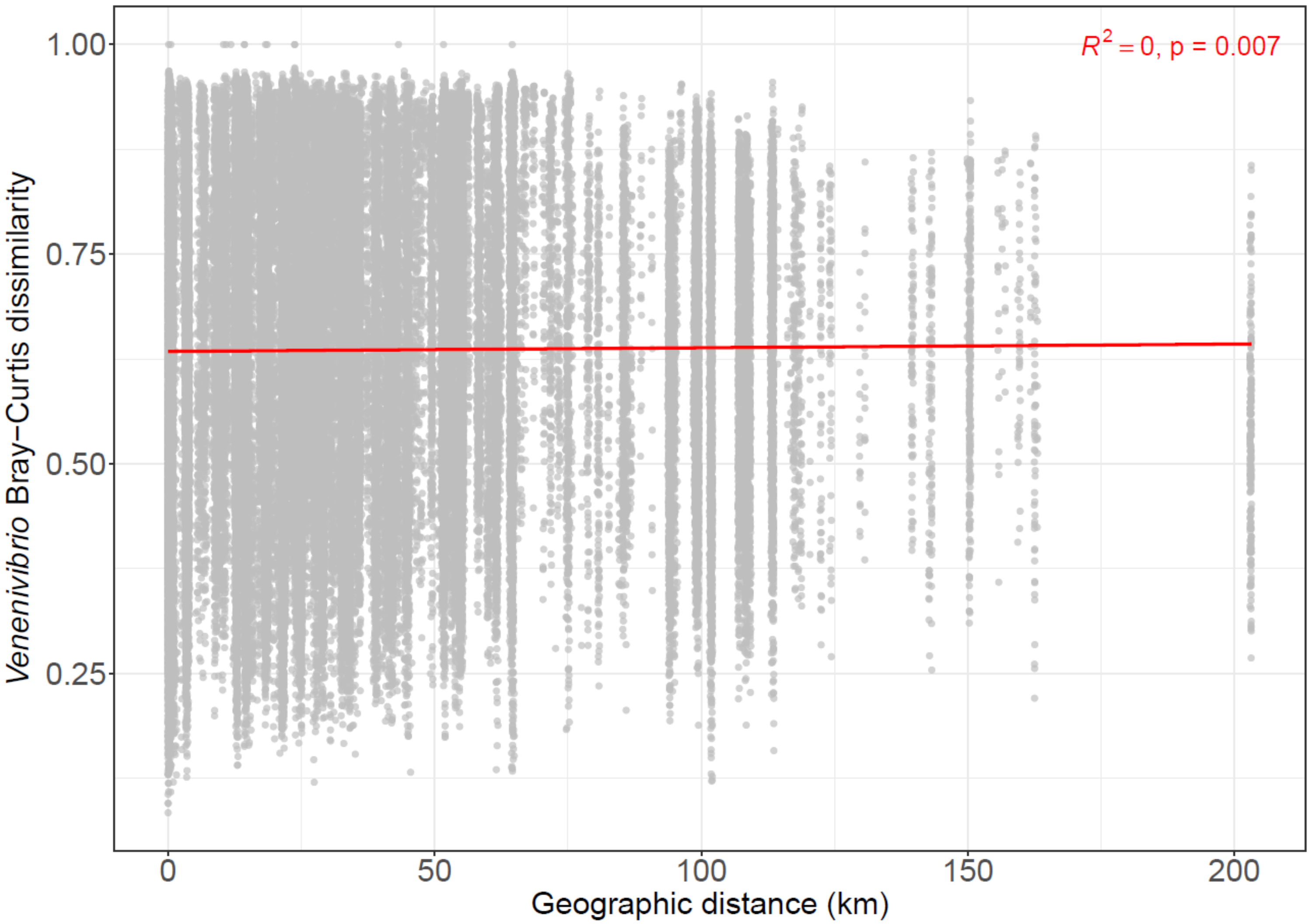

Figure S4. Distance-decay pattern of *Venenivibrio* populations in Aotearoa-New Zealand. Bray-Curtis dissimilarity was calculated between non-transformed *Venenivibrio* populations only (at operational taxonomic unit [OTU]-level) in geothermal springs (post-filtering, n=467) from across the TVZ, and plotted against pairwise geographic distance. A linear regression model was applied, highlighted in red (slope=4.36 x 10^{-5}).

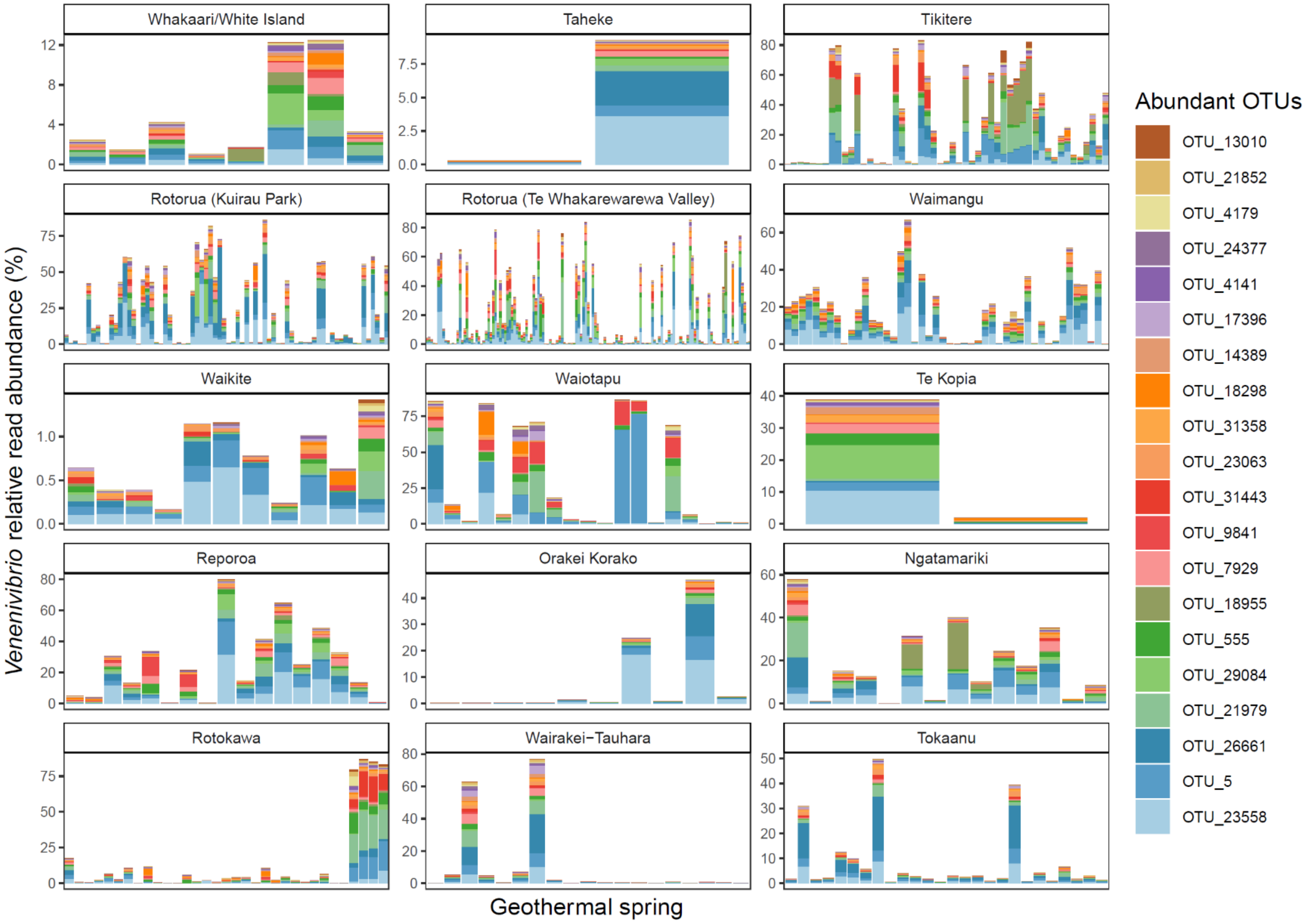

Figure S5. *Venenivibrio* relative read abundance per geothermal spring in Aotearoa-New Zealand. Springs that containing *Venenivibrio*-assigned reads (post filtering; *n*=467) are shown, split by respective geothermal field. Operational taxonomic units (OTUs) with the greatest read abundance (*n*=20) are represented by colour in each spring.

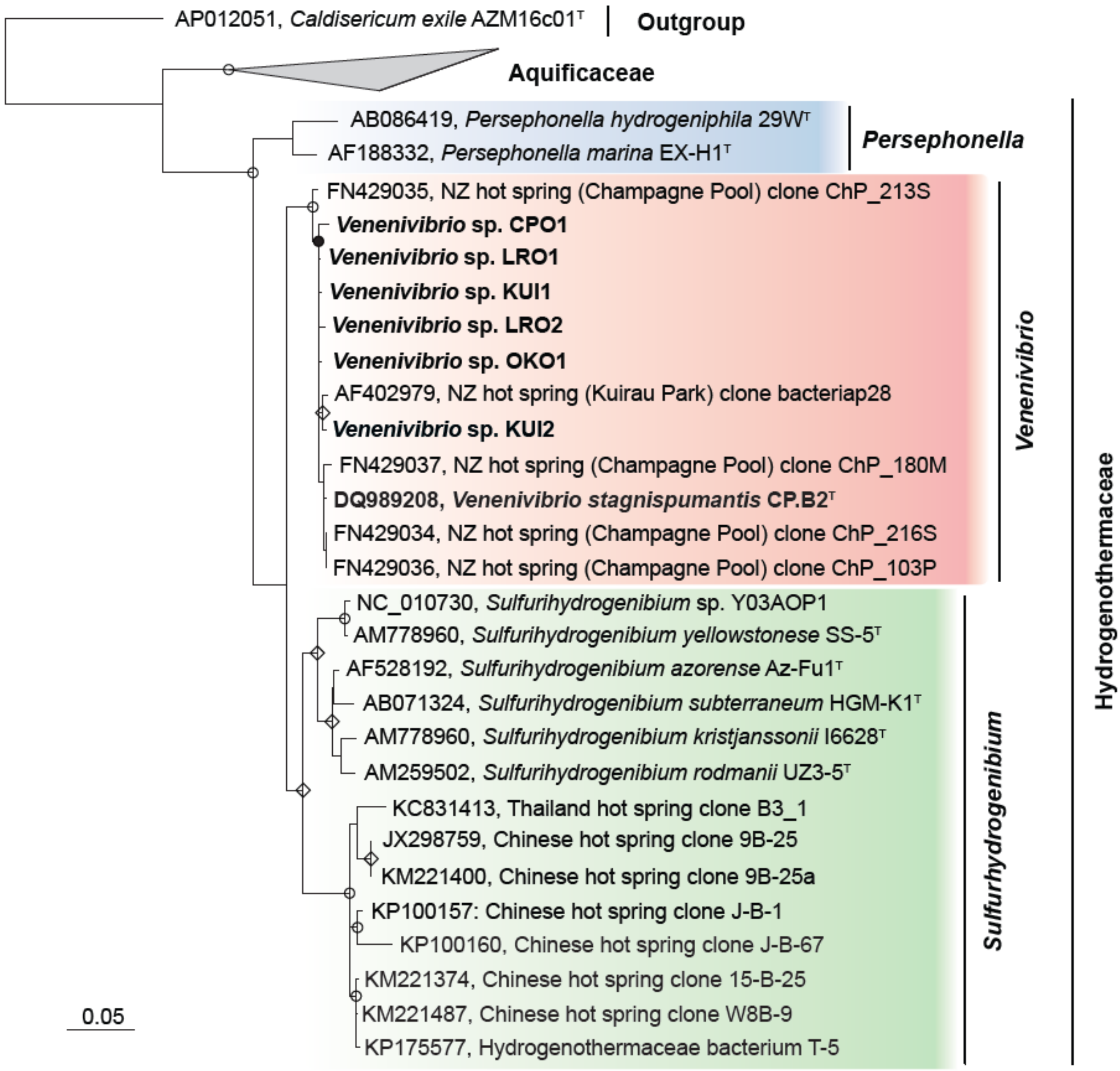

Figure S6. Phylogenetic tree of 16S rRNA gene sequences assigned to *Venenivibrio*. Maximum-likelihood quartet-puzzling phylogenetic tree showing the position of the type strain *Venenivibrio stagnispumantis* CP.B2^{T}, with near full-length environmental 16S rRNA gene clones that have been reported as belonging to or are closely related to the genus *Venenivibrio* from the SILVA database (SSU r138.1). Six new *Venenivibrio* strains recently isolated from the TVZ (CPO1, KUI1, KUI2, LRO1, LRO2 and OKO1), and type strains from closely related genera *Sulfurihydrogenibium* and *Persephonella* have also been included, with type strains from the family Aquificaceae collapsed for clarity. *Caldisericum exile* AZM16c01^{T} was used as an outgroup. Quartet-puzzling support values (10,000 resamples) are represented by the following symbols: [open circles] >90 %, [closed circles] >80 %, and [open diamonds] >70 % at each internal branch. Multifurcations are drawn where the support value for a bifurcation is <50 %. The scale bar represents 0.05 substitutions per nucleotide position.

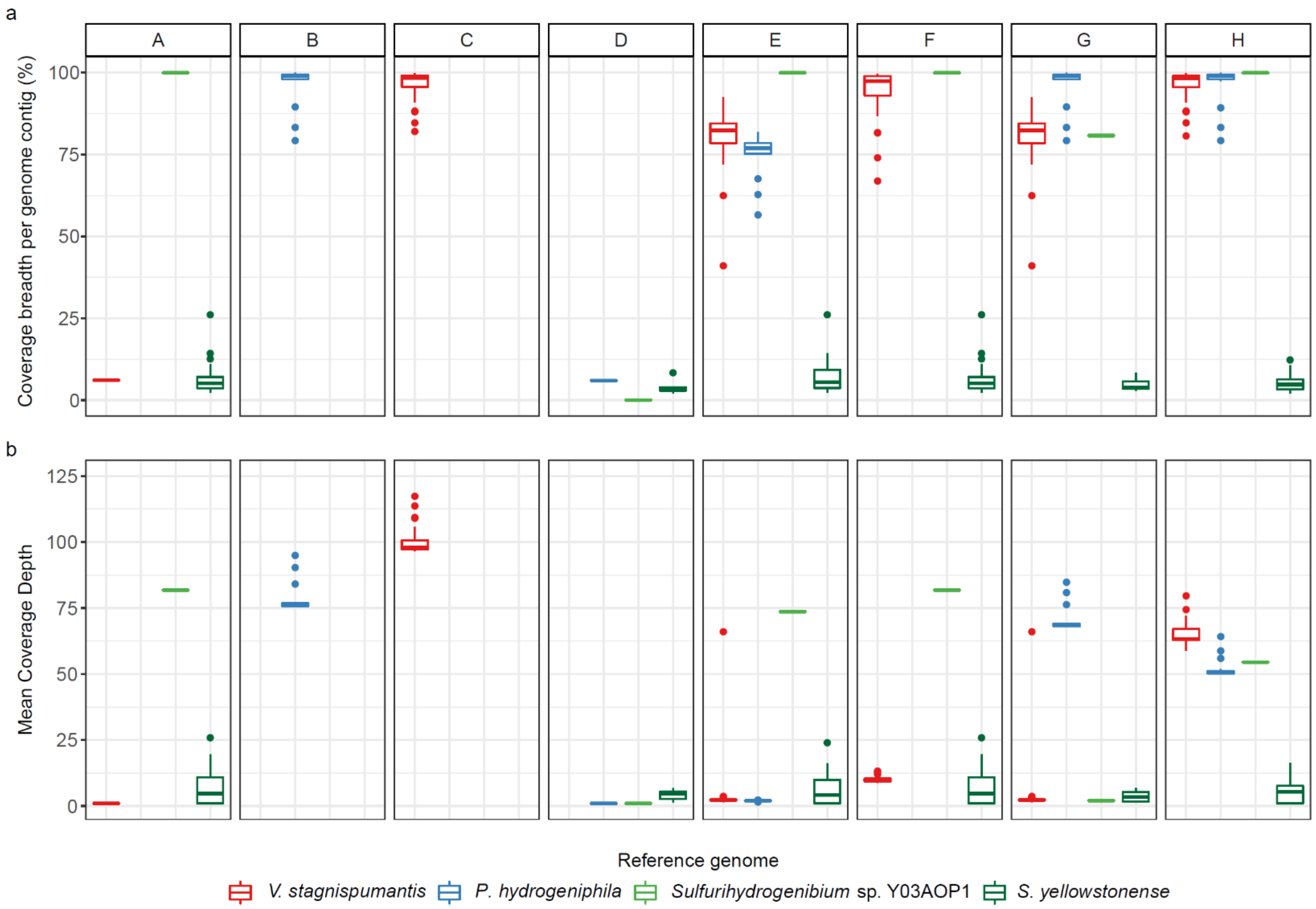

Figure S7. Hydrogenothermaceae genomes recovered from synthetic metagenomes. (a) Coverage breadth of reference genome contigs is displayed from alignment against eight mock metagenomic communities (A-H). **(b)** Average coverage depth of the reference genome contigs from the synthetic samples is shown. The four reference genomes used were *V. stagnispumantis* CP.B2^T (red, *n*=43 contigs, GOLD Analysis ID Ga0170441), *P. hydrogeniphila* 29W^T (blue, *n*=19 contigs, GenBank Accession GCA_900215515.1), *Sulfurihydrogenibium* sp. Y03AOP1 (light green, *n*=1 contig, GCA_000020325.1), and *Sulfurihydrogenibium yellowstonense* SS-5^T (dark green, *n*=228 contigs, GCF_000173615.1).

SUPPLEMENTARY REFERENCES

1. Power, J. F. *et al.* Draft genome sequence of *Venenivibrio stagnispumantis* CP.B2^T, isolated from Champagne Pool, Waiotapu, Aotearoa-New Zealand. *Microbiol. Resour. Announc.* **12**, e01074-22 (2023).
2. Hügler, M., Huber, H., Molyneaux, S. J., Vetriani, C. & Sievert, S. M. Autotrophic CO_2 fixation via the reductive tricarboxylic acid cycle in different lineages within the phylum Aquificae: evidence for two ways of citrate cleavage. *Environ. Microbiol.* **9**, 81–92 (2007).
3. Aoshima, M. & Igarashi, Y. A novel oxalosuccinate-forming enzyme involved in the reductive carboxylation of 2-oxoglutarate in *Hydrogenobacter thermophilus* TK-6. *Mol. Microbiol.* **62**, 748–759 (2006).
4. Steffens, L. *et al.* High CO_2 levels drive the TCA cycle backwards towards autotrophy. *Nature* **592**, 784–788 (2021).
5. Flamholz, A., Noor, E., Bar-Even, A., Liebermeister, W. & Milo, R. Glycolytic strategy as a tradeoff between energy yield and protein cost. *PNAS* **110**, 10039–10044 (2013).
6. Spaans, S. K., Weusthuis, R. A., van der Oost, J. & Kengen, S. W. M. NADPH-generating systems in bacteria and archaea. *Front. Microbiol.* **6**, 742 (2015).
7. Borisov, V. B., Gennis, R. B., Hemp, J. & Verkhovsky, M. I. The cytochrome *bd* respiratory oxygen reductases. *Biochim. Biophys. Acta* **1807**, 1398–1413 (2012).
8. Cleiss-Arnold, J. *et al.* Temporal transcriptomic response during arsenic stress in *Herminiimonas arsenicoxydans*. *BMC Genomics* **11**, 709 (2010).
9. Trumpower, B. L. The protonmotive Q cycle: energy transduction by coupling of proton translocation to electron transfer by the cytochrome *bc*1 complex. *J. Biol. Chem.* **265**, 11409–11412 (1990).
10. Boughanemi, S., Infossi, P., Giudici-Orticoni, M. T., Schoepp-Cothenet, B. & Guiral, M. Sulfite oxidation by the quinone-reducing molybdenum sulfite dehydrogenase SoeABC from the bacterium *Aquifex aeolicus*. *Biochim. Biophys. Acta - Bioenerg.* **1861**, 148279 (2020).
11. Koch, T. & Dahl, C. A novel bacterial sulfur oxidation pathway provides a new link between the cycles of organic and inorganic sulfur compounds. *ISME J.* **12**, 2479–2491 (2018).
12. Fekih, I. Ben *et al.* Distribution of arsenic resistance genes in prokaryotes. *Front. Microbiol.* **9**, 2473 (2018).
13. Cozen, A. E. *et al.* Transcriptional map of respiratory versatility in the hyperthermophilic crenarchaeon *Pyrobaculum aerophilum*. *J. Bacteriol.* **191**, 782–794 (2009).
14. Giloteaux, L. *et al.* Characterization and transcription of arsenic respiration and resistance genes during *in situ* uranium bioremediation. *ISME J.* **7**, 370–383 (2013).
15. Hu, M. *et al.* New arsenite oxidase gene (*aioA*) PCR primers for assessing arsenite-oxidizer diversity in the environment using high-throughput sequencing. *Front. Microbiol.* **12**, 691913 (2021).
16. Hamamura, N. *et al.* Linking microbial oxidation of arsenic with detection and phylogenetic analysis of arsenite oxidase genes in diverse geothermal environments. *Environ. Microbiol.* **11**, 421–431 (2009).
17. Lee, K. C. *et al.* The *Chthonomonas calidirosea* genome is highly conserved across geography and distinct chemical and microbial environments in the Taupō Volcanic Zone, New Zealand. *Appl. Environ. Microbiol.* **82**, 3572–3581 (2016).

18. Reysenbach, A. L. *et al.* Complete and draft genome sequences of six members of the Aquificales. *J. Bacteriol.* **191**, 1992–1993 (2009).
19. Hetzer, A., Morgan, H. W., McDonald, I. R. & Daughney, C. J. Microbial life in Champagne Pool, a geothermal spring in Waiotapu, New Zealand. *Extremophiles* **11**, 605–614 (2007).
20. Hetzer, A., McDonald, I. R. & Morgan, H. W. *Venenivibrio stagnispumantis* gen. nov., sp. nov., a thermophilic hydrogen-oxidizing bacterium isolated from Champagne Pool, Waiotapu, New Zealand. *Int. J. Syst. Evol. Microbiol.* **58**, 398–403 (2008).
21. Sunna, A. & Bergquist, P. L. A gene encoding a novel extremely thermostable 1,4-β-xylanase isolated directly from an environmental DNA sample. *Extremophiles* **7**, 63–70 (2003).
22. Childs, A. M., Mountain, B. W., O'Toole, R. & Stott, M. B. Relating microbial community and physicochemical parameters of a hot spring: Champagne Pool, Waiotapu, New Zealand. *Geomicrobiol. J.* **25**, 441–453 (2008).
23. Tamazawa, S., Takasaki, K., Tamaki, H., Kamagata, Y. & Hanada, S. Metagenomic and biochemical characterizations of sulfur oxidation metabolism in uncultured large sausage-shaped bacterium in hot spring microbial mats. *PLoS One* **7**, e49793 (2012).
24. Yeoh, Y. K. *et al.* The core root microbiome of sugarcanes cultivated under varying nitrogen fertilizer application. *Environ. Microbiol.* **18**, 1338–1351 (2016).
25. Grasby, S. E. *et al.* The Paint Pots, Kootenay National Park, Canada - a natural acid spring analogue for mars. *Can. J. Earth Sci.* **50**, 94–108 (2013).
26. Kaur, G., Mountain, B. W., Stott, M. B., Hopmans, E. C. & Pancost, R. D. Temperature and pH control on lipid composition of silica sinters from diverse hot springs in the Taupo Volcanic Zone, New Zealand. *Extremophiles* **19**, 327–344 (2015).
27. Oliverio, A. M. *et al.* The ecology and diversity of microbial eukaryotes in geothermal springs. *ISME J.* **12**, 1918–1928 (2018).
28. Power, J. F. *et al.* Microbial biogeography of 925 geothermal springs in New Zealand. *Nat. Commun.* **9**, 2876 (2018).
29. Martijn, J. *et al.* Confident phylogenetic identification of uncultured prokaryotes through long read amplicon sequencing of the 16S-ITS-23S rRNA operon. *Environ. Microbiol.* **21**, 2485–2498 (2019).
30. Massello, F. L. *et al.* Meta-analysis of microbial communities in hot springs: recurrent taxa and complex shaping factors beyond pH and temperature. *Microorganisms* **8**, 906 (2020).
31. Hedlund, B. P. *et al.* Isolation of diverse members of the Aquificales from geothermal springs in Tengchong, China. *Front. Microbiol.* **6**, 157 (2015).
32. Giovannelli, D. *et al.* Insight into the evolution of microbial metabolism from the deep-branching bacterium, *Thermovibrio ammonificans*. *Elife* **6**, e18990 (2017).
33. Dixit, S. *et al.* Bacterial diversity and CAZyme potential revealed in Pandanus rich thermal spring cluster of India: a non-cultivable 16S rRNA sequencing approach. *Front. Microbiol.* **12**, 760573 (2021).
34. Yabe, S., Kato, A., Hazaka, M. & Yokota, A. *Thermaerobacter composti* sp. nov., a novel extremely thermophilic bacterium isolated from compost. *J. Gen. Appl. Microbiol.* **55**, 323–328 (2009).
35. Isobe, K. *et al.* A simple and rapid GC/MS method for the simultaneous determination of gaseous metabolites. *J. Microbiol. Methods* **84**, 46–51 (2011).

36. Peng, X. & Jones, B. Rapid precipitation of silica (opal-A) disguises evidence of biogenicity in high-temperature geothermal deposits: case study from Dagunguo hot spring, China. *Sediment. Geol.* **257**–**260**, 45–62 (2012).
37. Gupta, R. S. & Lali, R. Molecular signatures for the phylum Aquificae and its different clades: proposal for division of the phylum Aquificae into the emended order Aquificales, containing the families Aquificaceae and Hydrogenothermaceae, and a new order Desulfurobacteriales. *Antonie van Leeuwenhoek, Int. J. Gen. Mol. Microbiol.* **104**, 349–368 (2013).
38. Härtig, C. *et al.* Chemolithotrophic growth of the aerobic hyperthermophilic bacterium *Thermocrinis ruber* OC 14/7/2 on monothioarsenate and arsenite. *FEMS Microbiol. Ecol.* **90**, 747–760 (2014).
39. Stott, M. B. & Taylor, M. W. Microbial ecology research in New Zealand. *N. Z. J. Ecol.* **40**, 12–28 (2016).
40. Stewart, L. C., Stucker, V. K., Stott, M. B. & Ronde, C. E. J. De. Marine-influenced microbial communities inhabit terrestrial hot springs on a remote island volcano. *Extremophiles* **22**, 687–698 (2018).
41. Stewart, L. C. *et al.* Interaction between ferruginous clay sediment and an iron-reducing hyperthermophilic *Pyrobaculum* sp. in a terrestrial hot spring. *FEMS Microbiol. Ecol.* **94**, fiy160 (2018).
42. François, D. X. *et al. Persephonella atlantica* sp. nov.: how to adapt to physico-chemical gradients in high temperature hydrothermal habitats. *Syst. Appl. Microbiol.* **44**, 126176 (2021).
43. Guo, L. *et al.* Temperature governs the distribution of hot spring microbial community in three hydrothermal fields, eastern Tibetan Plateau geothermal belt, western China. *Sci. Total Environ.* **720**, 137574 (2020).
44. Xiao, E. *et al.* The physiological response of *Arundo donax* and characteristics of anodic bacterial community in BE-CW systems: effects of the applied voltage. *Chem. Eng. J.* **380**, 122604 (2020).
