# Supplementary material for: A genus in the bacterial phylum Aquificota appears to be endemic to Aotearoa-New Zealand
Source: Nat Commun. 2024 Jan 2;15:179. doi: 10.1038/s41467-023-43960-2 (PMC10762115; doi:10.1038/s41467-023-43960-2)
Supplement: Supplementary file 3 — Description of Additional Supplementary Files [file 41467_2023_43960_MOESM3_ESM.pdf]

Description of Additional Supplementary Files:

Supplementary Data 1. Aquificota-assigned taxa in Aotearoa-New Zealand geothermal springs. Unfiltered data from the 1,000 Springs Project (Power *et al.*, 2018) shows total read abundance of each genus, mean relative abundance across all 925 springs with standard deviation, maximum relative abundance (Max) found in a single spring, the number of springs where each genus was found, percentage prevalence across the dataset, and the number of OTUs assigned to each genus.

Supplementary Data 2. Single linear regressions of Aquificota diversity against 46 environmental variables. Both OTU number and total read abundance in each spring were Aquificota was found (post filtering; n=783) were analysed against 46 physicochemical parameters using linear regression. Results are ordered by descending R^2 value (OTU number) with corresponding two-tailed p-value displayed. No transformations were performed on any parameter prior to analysis. The false discovery rate (FDR) Benjamini-Hochberg correction was applied to all p-values to correct for multiple comparisons (corrected p-value).

Supplementary Data 3. Multiple linear regression model of Aquificota OTU number against metadata. Multiple linear regression was performed between OTU number and all physicochemical variables from each spring where Aquificota reads were present (post filtering; n=783). Samples with missing or aberrant data were not included in this analysis (n=14). An Akaike information criterion (AIC) was applied to find the model of best fit, with the final R^2 value being 0.389 (p<0.001). Results are ordered by ascending two-tailed p-value. The false discovery rate (FDR) Benjamini-Hochberg correction was applied to all p-values to correct for multiple comparisons (corrected p-value).

Supplementary Data 4. Multiple linear regression model of Aquificota read abundance against metadata. Multiple linear regression was performed between Aquificota read abundance and all physicochemical variables from each spring where Aquificota reads were present (post filtering; n=783). Samples with missing or aberrant data were not included in the analysis (n=14). An Akaike information criterion (AIC) was applied to find the model of best fit, with the R^2 value for the final model being 0.358 (p<0.001). Results are ordered by ascending two-tailed p-value. The false discovery rate (FDR) Benjamini-Hochberg correction was applied to all p-values to correct for multiple comparisons (corrected p-value).

Supplementary Data 5. Correlations between Aquificota diversity and environmental variables. Both OTU number and read abundance in each spring (post filtering; n=783) was correlated against 46 physicochemical parameters using two-sided Pearson's and Spearman's coefficient. Due to missing or aberrant data, only 771, 781, and 782 springs were used to analyse conductivity, HCO_3^-, and H_2S, respectively. The false discovery rate (FDR) Benjamini-Hochberg correction was applied to all p-values. Results are ordered by descending Pearson's coefficient for correlations with OTU number.

Supplementary Data 6. Characteristics of *Venenivibrio*-assigned OTUs. OTUs (post filtering; n=99) are ordered by descending mean relative abundance across the original 925 geothermal springs

analysed. The number of springs where each OTU was found (Prevalence), standard deviation of the mean relative abundance (SD), the maximum relative abundance in an individual spring (Max), sequence identity to *V. stagnispumantis* CP.B2^T using NCBI's BLAST algorithm, and the corresponding Expect (E) value are also shown.

Supplementary Data 7. Single linear regressions of *Venenivibrio* OTU number and read abundance against 46 metadata. The physicochemical variables were measured from each spring where *Venenivibrio* was present (post filtering; *n*=467). Results of the linear regressions are ordered by descending R^2 value for OTU number with corresponding two-tailed *p*-value displayed. No transformations performed on any parameter prior to analysis. The false discovery rate (FDR) Benjamini-Hochberg correction was applied to all *p*-values to correct for multiple comparisons (corrected *p*-value).

Supplementary Data 8. Multiple linear regression model of *Venenivibrio* OTU number against metadata. Multiple linear regression was performed between *Venenivibrio* OTU number and all physicochemical variables measured from each spring where *Venenivibrio* was present (post filtering; *n*=467). Two samples with missing HCO_3^- and H_2S data were removed from the analysis. An Akaike information criterion (AIC) was also applied to find the model of best fit. Results are ordered by ascending two-tailed *p*-value. The R^2 value for the final model was 0.192 (p<0.001). The false discovery rate (FDR) Benjamini-Hochberg correction was applied to all *p*-values to correct for multiple comparisons (corrected *p*-value).

Supplementary Data 9. Multiple linear regression model of *Venenivibrio* read abundance against metadata. Multiple linear regression was performed between *Venenivibrio* read abundance and all physicochemical variables measured from each spring where *Venenivibrio* was present (post filtering; *n*=467). Two samples with missing HCO_3 and H_2S data were removed from the analysis. An Akaike information criterion (AIC) was also applied to find the model of best fit. with the R^2 value for the final model being 0.183 (p<0.001). Results are ordered by ascending two-tailed *p*-value. The false discovery rate (FDR) Benjamini-Hochberg correction was applied to all *p*-values to correct for multiple comparisons (corrected *p*-value).

Supplementary Data 10. Correlations between *Venenivibrio* diversity and environmental variables. Both OTU number and read abundance in each spring where *Venenivibrio* was found (post filtering; *n*=467) were correlated against 46 physicochemical parameters using two-sided Pearson's and Spearman's coefficient. Due to missing or aberrant data, only 455, 465, and 466 springs were used to analyse conductivity, HCO_3^- and H_2S, respectively. The false discovery rate (FDR) Benjamini-Hochberg correction was applied to all *p*-values. Results are ordered by descending Pearson's coefficient for correlations with OTU number.

Supplementary Data 11. Predicted function and annotation source of the *V. stagnispumantis* CP.B2^T genome. Genome assembly of the type strain was deposited in the Genomes Online Database (GOLD Analysis ID Ga0311387) and GenBank (GCF_026108055.1), with annotation performed using the Integrated Microbial Genomes platform v4.16.4 (IMG Genome ID 2799112217). Genes are ordered by predicted pathway or metabolism.

Supplementary Data 12. Growth tolerances of *V. stagnispumantis* CP.B2^T. Four growth characteristics of CP.B2^T were reanalysed to clarify widespread distribution of the genus in Aotearoa-New Zealand. These results were compared to findings from the original characterisation (Hetzer *et al.*, 2008). Growth was also tested in Obsidian Pool (OP) spring water, from Yellowstone National Park, USA.

Supplementary Data 13. 16S rRNA gene sequences assigned to the genus *Venenivibrio* in the SILVA SSU r138.1 database. Pairwise sequence similarity to *V. stagnispumantis* CP.B2^T is shown.

Supplementary Data 14. Search for *Venenivibrio* in the IMNGS database. The 16S rRNA gene of *V. stagnispumantis* CP.B2^T was queried against the full database of SRA amplicon samples (*n*=500,048) and the number of reads at 95, 97, and 99 % sequence similarity are shown. Sample size, description, and country of origin are also presented, along with the corresponding OTU for each read. Samples are ordered by decreasing percent identity to *V. stagnispumantis* CP.B2^T.

Supplementary Data 15. *Venenivibrio* search results from the Sequence Read Archive (SRA). Both amplicon and metagenomic samples in SRA were screened for similarity to the 16S rRNA gene sequence of *V. stagnispumantis* CP.B2^T (tax_id=407997), using the STAT tool in the Google Cloud BigQuery platform. Only samples with k-mer counts >25 are shown.

Supplementary Data 16. Geothermal spring metagenomes from the Taupō Volcanic Zone, Aotearoa-New Zealand, with associated metadata and *V. stagnispumantis* read abundance. Sixteen metagenomic samples from ten geothermal springs were screened for the presence of *Venenivibrio*, with read abundance counts and taxonomic classification performed using Kraken2. Sample characteristics presented are location details, description, temperature, and pH. Samples are ordered by descending relative abundance of *V. stagnispumantis*. Metagenomes deposited in the Aotearoa Genomic Data Repository (AGDR) can be found under the Project ID TAONGA-AGDR00025 at https://doi.org/10.57748/vpk8-zp44. Samples NZ1 and NZ8 can be found in the Genomes Online Database (GOLD) at https://gold.jgi.doe.gov/. All other samples are in the Sequence Read Archive (SRA) at https://www.ncbi.nlm.nih.gov/sra.

Supplementary Data 17. *Venenivibrio* read abundance in global spring metagenomes and associated metadata. Twenty-one samples returned traces of *V. stagnispumantis* CP.B2^T from community characterisation of 188 metagenomes. The samples are ordered by descending read number that assigned to the genus, with percentage of the whole community also shown. Samples that were subsequently aligned to Hydrogenothermaceae reference genomes (Supplementary Data 18) are highlighted.

Supplementary Data 18. Alignment of metagenomic samples to Hydrogenothermaceae genomes. Percentage of total genome coverage breadth per sample is shown, along with coverage depth, for each of the four genomes used in the alignment. Local metagenomes to Aotearoa-New Zealand are sample IDs CPp, P1.0019, NZ1, and P2.0050. The remaining six metagenomes are globally sourced.

Supplementary Data 19. Metagenome-assembled genomes (MAGs). Four local (CPp, NZ1, P1.0019, and P2.0050) and two global (DRR163687 and DRR163686) metagenomic samples were *de novo*

assembled and binned into MAGs. Average nucleotide identity (ANI) was confirmed to *V. stagnispumantis* CP.B2^T. Bins are ordered by descending ANI. MAGs deposited in the Aotearoa Genomic Data Repository (AGDR) can be found under the Project ID TAONGA-AGDR00025 at https://doi.org/10.57748/vpk8-zp44.

Supplementary Data 20. Synthetic metagenomes composition and classification. The makeup of the mock communities (sample A-H) is presented in the first column per genome, with the percentage classified using Kraken2 in the second column.

Supplementary Data 21. Alignment of synthetic metagenomic samples to Hydrogenothermaceae genomes. Average coverage breadth and depth are shown for contigs from each sample across the four genomes used in the alignment.
