# Supplementary material for: A genus in the bacterial phylum Aquificota appears to be endemic to Aotearoa-New Zealand
Source: Nat Commun. 2024 Jan 2;15:179. doi: 10.1038/s41467-023-43960-2 (PMC10762115; doi:10.1038/s41467-023-43960-2)
Supplement: Supplementary file 5 — Reporting Summary [file 41467_2023_43960_MOESM5_ESM.pdf]

nature portfolio

Corresponding author(s): Matthew Stott
S. Craig Cary

Last updated by author(s): Matthew Stott 08/Nov/2023

Reporting Summary

Nature Portfolio wishes to improve the reproducibility of the work that we publish. This form provides structure for consistency and transparency in reporting. For further information on Nature Portfolio policies, see our Editorial Policies and the Editorial Policy Checklist.

Statistics

For all statistical analyses, confirm that the following items are present in the figure legend, table legend, main text, or Methods section.

n/a	Confirmed	
☐	☒	The exact sample size (*n*) for each experimental group/condition, given as a discrete number and unit of measurement
☐	☒	A statement on whether measurements were taken from distinct samples or whether the same sample was measured repeatedly
☐	☒	The statistical test(s) used AND whether they are one- or two-sided<br>*Only common tests should be described solely by name; describe more complex techniques in the Methods section.*
☐	☒	A description of all covariates tested
☐	☒	A description of any assumptions or corrections, such as tests of normality and adjustment for multiple comparisons
☐	☒	A full description of the statistical parameters including central tendency (e.g. means) or other basic estimates (e.g. regression coefficient) AND variation (e.g. standard deviation) or associated estimates of uncertainty (e.g. confidence intervals)
☐	☒	For null hypothesis testing, the test statistic (e.g. *F*, *t*, *r*) with confidence intervals, effect sizes, degrees of freedom and *P* value noted<br>*Give P values as exact values whenever suitable.*
☒	☐	For Bayesian analysis, information on the choice of priors and Markov chain Monte Carlo settings
☒	☐	For hierarchical and complex designs, identification of the appropriate level for tests and full reporting of outcomes
☐	☒	Estimates of effect sizes (e.g. Cohen's *d*, Pearson's *r*), indicating how they were calculated

Our web collection on statistics for biologists contains articles on many of the points above.

Software and code

Policy information about availability of computer code

Data collection	TerrestialMetagenomeDB release 2.0<br>SRA Tools v2.9.3
Data analysis	The following is a list of all software and versions used in data analysis:<br>USEARCH v7<br>QIIME v1.9<br>RDP Classifier v2.2<br>R v4.0.3<br>phyloseq v1.32<br>ggplot2 v3.3.2<br>ggmap v3.0.0<br>vegan v2.5-6<br>IMG Annotation Pipeline v4.16.4<br>BLASTN v2.13.0<br>STAT v2.11.2<br>SINA v1.2.11<br>IMNGS v1.0 build 2105<br>IMG/M v6.0<br>redbiom v0.3.5

ARB LTP_09_2021
Kraken2 v2.0.8
bowtie2 v2.3.5.1
samtools v1.10
bedtools v2.29.2
ATLAS v2.6a3
MEGAHIT v1.2.9
MetaBAT 2 v2.10
FastANI v1.32
CheckM v1.0.5
InSilicoSeq v1.5.1
Seqtk-1.3 r106
SpeciesTree v2.2.0
TimeTree v5

In addition, all custom R scripts used for data analyses and figures are available at https://gitlab.com/morganlab/collaboration-1000Springs/1000Springs in the venenivibrio_manuscript directory.

For manuscripts utilizing custom algorithms or software that are central to the research but not yet described in published literature, software must be made available to editors and reviewers. We strongly encourage code deposition in a community repository (e.g. GitHub). See the Nature Portfolio guidelines for submitting code & software for further information.

Data

Policy information about availability of data

All manuscripts must include a data availability statement. This statement should provide the following information, where applicable:

- Accession codes, unique identifiers, or web links for publicly available datasets
- A description of any restrictions on data availability
- For clinical datasets or third party data, please ensure that the statement adheres to our policy

The 925 raw amplicon sequences analysed in this manuscript from the 1,000 Springs Project can be found under the study accession PRJEB24353. Shotgun sequences of the V. stagnispumantis CP.B2T genome have been deposited at DDBJ/ENA/GenBank under accession JAPEIW010000000, with the assembled genome deposited into Genomes Online Database (GOLD Analysis ID Ga0311387). Associated annotations are available at Integrated Microbial Genomes system (IMG Taxon ID 2799112217) and GenBank (GCA_026108055.1). Microorganisms and the ecosystems with which they interact can be of cultural significance to Māori (the Indigenous People of Aotearoa New Zealand). These microbial ecosystems and the knowledge (mātauranga) associated with these systems can be considered taonga (a treasure). Additionally, under the Treaty of Waitangi, Māori have the right to retain control over data derived from species over which they hold kaitiakitanga (guardianship) and mana whenua (customary rights). For this reason, the Aotearoa-New Zealand-associated MAGs (Supplementary Data 19) have been uploaded into the New Zealand-located Aotearoa Genomic Data Repository (www.data.agdr.org.nz). Requests for access to these data can be initiated under Project ID TAONGA-AGDR00025 (https://doi.org/10.57748/vpk8-zp44) and will be granted on the recommendation of the iwi (extended kinship group) holding mana whenua over these data. The Aotearoa Genomic Data Repository (AGDR) has been recently described in a publication110. Caveats for data use are defined by mana whenua, but broadly, data cannot be on-shared or used for commercial purposes without permission, and there are no authorship requirements. For any additional information, please contact the corresponding authors of this manuscript. The 16S rRNA gene sequence and metagenomic search results (Supplementary Data 13-15), and local and global metagenomes analysed (Supplementary Data 16-17) are available as Supplementary Data files.

The following publicly-available DNA sequence databases were also screened for evidence of Venenivibrio:
1. NCBI's Nucleotide Collection (accessed 23/Jul/2021)
2. NCBI's Sequence Read Archive (accessed 06/Dec/2021)
3. SILVA SSU Database r138.1 (accessed 25/Mar/2022)
4. Ribosomal Database Project v11_6 (accessed 25/May/2021)
5. Greengenes Database v13_8 (accessed 02/Jul/2021)
6. Integrated Microbial Next-Generation Sequencing Platform v1.0 build 2105 (accessed 21/May/2021)
7. Integrated Microbial Genomes & Metagenomes system v6.0 (accessed 21/Jul/2021)
8. Earth Microbiome Project Release 1 (accessed 22/Jun/2021)
9. Qiita Database v2021.5 (accessed 05/Jul/2021)

Research involving human participants, their data, or biological material

Policy information about studies with human participants or human data. See also policy information about sex, gender (identity/presentation), and sexual orientation and race, ethnicity and racism.

Reporting on sex and gender	N/A
Reporting on race, ethnicity, or other socially relevant groupings	N/A
Population characteristics	N/A
Recruitment	N/A
Ethics oversight	N/A

Note that full information on the approval of the study protocol must also be provided in the manuscript.

Field-specific reporting

Please select the one below that is the best fit for your research. If you are not sure, read the appropriate sections before making your selection.

☐ Life sciences ☐ Behavioural & social sciences ☒ Ecological, evolutionary & environmental sciences

For a reference copy of the document with all sections, see nature.com/documents/nr-reporting-summary-flat.pdf

Life sciences study design

All studies must disclose on these points even when the disclosure is negative.

Sample size	N/A
Data exclusions	N/A
Replication	N/A
Randomization	N/A
Blinding	N/A

Behavioural & social sciences study design

All studies must disclose on these points even when the disclosure is negative.

Study description	N/A
Research sample	N/A
Sampling strategy	N/A
Data collection	N/A
Timing	N/A
Data exclusions	N/A
Non-participation	N/A
Randomization	N/A

Ecological, evolutionary & environmental sciences study design

All studies must disclose on these points even when the disclosure is negative.

Study description	This study investigated the apparent endemism of a thermophilic bacterial genus, Venenivibrio (family Hydrogenothermaceae), to New Zealand. We used national and international datasets (outlined in the Data section above) to demonstrate that while the genus is abundant and broadly distributed across New Zealand, it is not detectable outside New Zealand. We then investigated the possible factors (genomics, physiological and environmental) that may have lead to this apparent endemism. The design structure of the quantitative data analysed in this study was factorial, with no treatment factors or interactions. This included 46 physicochemical parameters and DNA measured from the water columns of 925 geothermal springs and subsequent DNA sequencing. Amplicon sequencing reads were normalised to 9,500 reads per sample prior to extracting diversity information (i.e., number of operational taxonomic units and respective abundance levels per sample). Replicates in triplicate were taken for DNA extractions and all physiological experiments in re-characterising the type strain for Venenivibrio, V. stagnispumantis CP.B2. Two separate DNA sequencing of the CP.B2 genome and subsequent annotations were interpreted for genomic capability of the type strain. Sixteen local and 188 global metagenomes were also screened for DNA sequence similarity to Venenivibrio, with phylogenomics of all available Hydrogenothermaceae genomes performed to confirm evolutionary relatedness to Venenivibrio.
Research sample	Research samples were from four sources:<br>1. The dataset from the 1,000 Springs Project (Power et al., 2018; Nat Comms). This was the 16S rRNA gene sequence amplicon and physicochemical datasets from 925 geothermal springs in the Taupo Volcanic Zone, New Zealand that was used to demonstrate the presence and abundance of both phylum Aquificota and genus Venenivibrio in New Zealand. This dataset was chosen based on findings published in Power et al., 2018.<br>2. Sequenced Hydrogenothermaceae genomes, either sequenced de novo, or downloaded from NCBI RefSeq. Genomes were selected based on sequence similarity to Venenivibrio stagnispumantis strain CP.B2.

	3. Screening of a diverse set of publicly available databases for metagenomes and 16S rRNA gene sequences related to Venenivibrio (a thorough description of the criteria for screening is presented in the Methods). Every available DNA sequence database (outlined in the Data section above) was screened to provide the most extensive search possible.<br>4. Cultivation and phenotypic characterisation of the Venenivibrio type strain CP.B2, sourced from the original research group (Thermophile Research Unit, University of Waikato, NZ) that isolated the strain (Hetzer et al., 2008). No manipulations of the organism were performed.
Sampling strategy	We did not statistically determine our sample size. Rather we used all data available to us. These were:<br>(i) the 1000 Springs project which is a comprehensive microbial community dataset for the majority of hot springs in New Zealand (this reflects all comparable microbiological data for all geothermal springs in NZ (n=925 springs)); and<br>(ii) we screened all global datasets we were able to access for metagenomic and 16 rRNA gene sequence signatures. This represented ~ 971,117 samples, 26.7 billion 16S rRNA gene sequences, and 12.2 petabytes of sequence data
Data collection	Data collection for the physiological characterisation of the type strain was undertaken according to the guidelines dictated by the International Code of Nomenclature of Prokaryotes. This was performed by Matthew Stott and Holly Welford, with results observed by phase contrast microscopy and recorded in Microsoft Office word documents and excel spreadsheets. The rest of the data (16S rRNA gene amplicon sequencing, metagenomic sequencing, and genomic sequencing) were collected computationally by Jean Power, Daniel Hudson, and Xochitl Morgan using SRA Tools v2.9.3, InSilicoSeq v1.5.1, or manually downloaded from DNA sequence repositories. Physicochemical parameters were downloaded from the 1,000 Springs Project database (Power et al., 2018).
Timing and spatial scale	International datasets were screened between May 2021 and March 2022. The exact dates for each database are provided in the Methods, with each dataset screened once. All available global samples (n=971,11) were from multiple locations across the world where microbial DNA sequences have been collected and stored in international databases. Local amplicon (n=925) and metagenomic (n=16) DNA sequencing samples were taken from geothermal springs located in the Taupo Volcanic Zone, New Zealand. Physiological results from the type strain CP.B2 re-characterisation were collected after seven days incubation at 70 degree Celsius as this represented stationary phase of growth for the bacterium.
Data exclusions	The only data excluded in this study was when we normalised the 16S rRNA gene sequence dataset (n=9,500 sequencing reads) to allow for effective comparison of communities. Amplicon sequences were randomly removed to the set number of reads.
Reproducibility	Physiological testing of the bacterial type strain was undertaken in triplicate. All attempts at replication were successful.
Randomization	No randomisation was performed as the majority of data analyses were the presence/absence of the type strain in datasets.
Blinding	No blinding was required as analysis was primarily determining the presence/absence of the type strain in datasets.

Did the study involve field work? ☐ Yes ☒ No

Reporting for specific materials, systems and methods

We require information from authors about some types of materials, experimental systems and methods used in many studies. Here, indicate whether each material, system or method listed is relevant to your study. If you are not sure if a list item applies to your research, read the appropriate section before selecting a response.

Materials & experimental systems

n/a	Involved in the study	
☒	☐	Antibodies
☒	☐	Eukaryotic cell lines
☒	☐	Palaeontology and archaeology
☒	☐	Animals and other organisms
☒	☐	Clinical data
☒	☐	Dual use research of concern
☒	☐	Plants

Methods

n/a	Involved in the study	
☒	☐	ChIP-seq
☒	☐	Flow cytometry
☒	☐	MRI-based neuroimaging

Antibodies

Antibodies used	N/A
Validation	N/A
